# Prediction of COVID-19 deterioration in high-risk patients at diagnosis: an early warning score for advanced COVID-19 developed by machine learning

**DOI:** 10.1007/s15010-021-01656-z

**Published:** 2021-07-19

**Authors:** Carolin E. M. Jakob, Ujjwal Mukund Mahajan, Marcus Oswald, Melanie Stecher, Maximilian Schons, Julia Mayerle, Siegbert Rieg, Mathias Pletz, Uta Merle, Kai Wille, Stefan Borgmann, Christoph D. Spinner, Sebastian Dolff, Clemens Scherer, Lisa Pilgram, Maria Rüthrich, Frank Hanses, Martin Hower, Richard Strauß, Steffen Massberg, Ahmet Görkem Er, Norma Jung, Jörg Janne Vehreschild, Hans Stubbe, Lukas Tometten, Rainer König, The LEOSS Study group, Lukas Tometten, Siegbert Rieg, Uta Merle, Kai Wille, Stefan Borgmann, Christoph Spinner, Sebastian Dolff, Maria Madeleine Rüthrich, Frank Hanses, Martin Hower, Richard Strauß, Murat Akova, Norma Jung, Michael von Bergwelt-Baildon, Maria Vehreschild, Beate Grüner, Martina Haselberger, Nora Isberner, Christiane Piepel, Kerstin Hellwig, Dominic Rauschning, Lukas Eberwein, Björn Jensen, Claudia Raichle, Gabriele Müller-Jörger, Sven Stieglitz, Thomas Kratz, Christian Degenhardt, Anette Friedrichs, Robert Bals, Susanne Rüger, Katja With, Katja Rothfuss, Siri Goepel, Jacob Nattermann, Sabine Jordan, Jessica Rüddel, Janina Trauth, Gernot Beutel, Ozlem Altuntas Aydin, Milena Milovanovic, Michael Doll, Jörg Janne Vehreschild, Lisa Pilgram, Melanie Stecher, Carolin E. M. Jakob, Maximilian Schons, Annika Claßen, Sandra Fuhrmann, Susana Nunes de Miranda, Bernd Franke, Nick Schulze, Fabian Prasser, Martin Lablans

**Affiliations:** 1 Department I of Internal Medicine, University Hospital of Cologne, University of Cologne, Cologne, Germany; 2 German Center for Infection Research, Partner Site Bonn-Cologne, Cologne, Germany; 3 Department of Medicine II, University Hospital, LMU Munich, Campus Großhadern, Marchioninistr. 15, Munich, 81377 Germany; 4 Institute for Infectious Diseases and Infection Control, RG Systemsbiology, Jena University Hospital, Kollegiengasse 10, 07743 Jena, Germany; 5 Division of Infectious Diseases, Department of Medicine II, Faculty of Medicine, Medical Center-University of Freiburg, University of Freiburg, Freiburg, Germany; 6 Department of Internal Medicine IV, University Hospital Heidelberg, Heidelberg, Germany; 7 Johannes Wesling Hospital Minden, University Clinic for Hematology, Oncology, Hemostaseology and Palliative Care, University of Bochum, Bochum, Germany; 8 Department of Infectious Diseases and Infection Control, Ingolstadt Hospital, Ingolstadt, Germany; 9 Department of Internal Medicine II, School of Medicine, University Hospital Rechts Der Isar, Technical University of Munich, Munich, Germany; 10 Department of Infectious Diseases, West German Centre of Infectious Diseases, University Hospital Essen, Essen, Germany; 11 Department of Medicine I, University Hospital, LMU Munich, Munich, Germany; 12 Department of Internal Medicine, Hematology and Oncology, Goethe University Frankfurt, Frankfurt am Main, Germany; 13 Department of Internal Medicine II, Hematology and Medical Oncology, University Hospital Jena, Jena, Germany; 14 Emergency Department, University Hospital Regensburg, Regensburg, Germany; 15 Department of Infection Prevention and Infectious Diseases, University Hospital Regensburg, Regensburg, Germany; 16 Department of Pneumology, Infectious Diseases and Intensive Care, Klinikum Dortmund gGmbH, Dortmund, Germany; 17 Department of Medicine 1, University Hospital Erlangen, Erlangen, Germany; 18 Department of Infectious Diseases and Clinical Microbiology, Hacettepe University Faculty of Medicine, Ankara, Turkey

**Keywords:** COVID-19, Machine learning, Predictive model, Advanced stage, Complicated stage, LEOSS

## Abstract

**Purpose:**

While more advanced COVID-19 necessitates medical interventions and hospitalization, patients with mild COVID-19 do not require this. Identifying patients at risk of progressing to advanced COVID-19 might guide treatment decisions, particularly for better prioritizing patients in need for hospitalization.

**Methods:**

We developed a machine learning-based predictor for deriving a clinical score identifying patients with asymptomatic/mild COVID-19 at risk of progressing to advanced COVID-19. Clinical data from SARS-CoV-2 positive patients from the multicenter Lean European Open Survey on SARS-CoV-2 Infected Patients (LEOSS) were used for discovery (2020-03-16 to 2020-07-14) and validation (data from 2020-07-15 to 2021-02-16).

**Results:**

The LEOSS dataset contains 473 baseline patient parameters measured at the first patient contact. After training the predictor model on a training dataset comprising 1233 patients, 20 of the 473 parameters were selected for the predictor model. From the predictor model, we delineated a composite predictive score (SACOV-19, Score for the prediction of an Advanced stage of COVID-19) with eleven variables. In the validation cohort (*n* = 2264 patients), we observed good prediction performance with an area under the curve (AUC) of 0.73 ± 0.01. Besides temperature, age, body mass index and smoking habit, variables indicating pulmonary involvement (respiration rate, oxygen saturation, dyspnea), inflammation (CRP, LDH, lymphocyte counts), and acute kidney injury at diagnosis were identified. For better interpretability, the predictor was translated into a web interface.

**Conclusion:**

We present a machine learning-based predictor model and a clinical score for identifying patients at risk of developing advanced COVID-19.

**Supplementary Information:**

The online version contains supplementary material available at 10.1007/s15010-021-01656-z.

## Introduction

In December 2019, a cluster of severe pneumonia occurred in the city of Wuhan, China. The causative pathogen was identified as a new betacoronavirus [1]. It was later named the Severe Acute Respiratory Syndrome Coronavirus-2 (SARS-CoV-2) and the infectious disease was termed coronavirus disease 2019 (COVID-19) [2]. As of September 2020, more than 32 million infections were reported worldwide and over 970,000 people had died [3]. Course and outcome of patients with COVID-19 are heterogeneous. While most SARS-CoV-2 infected patients are asymptomatic or exhibit mild symptoms, some deteriorate to the complicated stage and require medical treatment and hospitalization. COVID-19 symptoms can deteriorate within hours of hospital admission prompting need for oxygen supply or transfer to the intensive care unit [4, 5]. Hence, identifying patients at this early stage of the disease is of paramount importance in medical decision-making regarding follow-up, hospitalization, and decision for medical treatment.

Many studies investigated predictors for progression to critical COVID-19, which was defined as admission to an intensive care unit (ICU) or need for mechanical ventilation [6–10]. However, predictors for a COVID-19 deterioration causing oxygen therapy, have been rarely studied so far [11–13]. Depending on the clinical perspective, this stage of the disease is denoted in the literature as severe, but not critical [14–16] or moderate, but not severe [11, 13]. To avoid misinterpretations of our analysis, in the following, we use the term advanced COVID-19 disease stage for this stage of the disease and this was used as our endpoint to be predicted. Patients presenting with asymptomatic SARS-CoV-2 infection or mild COVID-19 who are at risk for clinical deterioration benefit from close monitoring, swift medication and supportive measurements [17]. Further, patients at risk may benefit from early therapeutic agents for COVID-19 [14, 16]. In addition, due to the high prevalence of long-term COVID-19 symptoms and the association of severity of COVID-19 and severity of long-term COVID-19 symptoms [18–20], the need for medical interventions avoiding COVID-19 disease progression in patients at risk is further emphasized.

Here, we present a predictor and score (SACOV-19, Score for the prediction of an Advanced disease stage of COVID-19) resulting from a robust risk-stratification algorithm to assess if a patient is at risk of developing the advanced COVID-19 disease stage, based on data available at the day of the first positive SARS-CoV-2 test. By identifying patients at risk with a high probability for advanced COVID-19, our score aims at supporting clinical decision making for these patients presenting with asymptomatic SARS-CoV-2 infection or mild COVID-19. A low predicted risk could support out-patient management. A high predicted risk could promote close follow-up, hospitalization or enter risk–benefit assessments regarding medical treatment.

The algorithm and SACOV-19 were developed using state-of-the-art machine learning methods and based on patient variables from the study cohort of the Lean European Open Survey on SARS-CoV-2 Infected Patients (LEOSS). LEOSS is a large multicenter cohort of medically supervised patients with predominant hospital contact [21]. The algorithm and SACOV-19 were assessed by a temporal validation using the LEOSS data. The algorithm is implemented in a browser-based web application enabling straightforward usage of our predictor in future clinical studies and to make it accessible to the research community.

## Methods

### Patient population and data collection

The prediction algorithm and SACOV-19 were developed and validated on patient data from LEOSS, the multicenter international COVID-19 registry comprising over 7000 patients collected in more than 100 study sites (http://www.leoss.net). Inclusion criteria for LEOSS were a laboratory confirmed SARS-CoV-2 infection from any respiratory material and clinical information available on follow-up until the end of the treatment (recovery or death). The day of the first SARS-CoV-2 diagnosis was referred to as the baseline time point. Documentation in LEOSS was performed retrospectively and anonymous. All patients’ variables, and also rational data such as age, BMI or laboratory data was collected in categories. Due to the anonymous data collection, written informed consent of the participants was waived by the respective ethics committees. For patients, recruited in Turkey, informed consent was obtained from the participants upon request of the national ethics committee. To reduce the risk of re-identification, the data was additionally anonymized using the principles used for the LEOSS Public Use File (PUF) we described earlier [22]. Approval for LEOSS data collection and analysis was obtained by the applicable local ethics committees of all participating centers and registered at the German Clinical Trials Registry (DRKS, No. S00021145).

In this study, patients were included who were asymptomatic or exhibited mild symptoms (symptoms of the upper respiratory tract, fever, nausea, emesis or diarrhea) at baseline.

Progression to a complicated or severe stage of COVID-19 during medical consultation/observational period was set as the endpoint (denoted as advanced COVID-19 stage). Since COVID-19 is a multi-organ disease, any incident organ failure during the disease was considered a complication. It was defined by the occurrence of at least one of the following symptoms during the observational period (complicated or critical COVID-19 stage according to LEOSS criteria [21]): need for new oxygen supplementation due to clinical deterioration, oxygen saturation (SO_2_) at room air < 90%, partial pressure of oxygen (PaO_2_) at room air < 70 mmHg, clinically meaningful increase of oxygen supplementation compared to prior oxygen home therapy, increase of aspartate aminotransferase (AST) or alanine aminotransferase (ALT) > 5 × ULN (upper limit of normal), new cardiac arrhythmia, new pericardial effusion > 1 cm or new heart failure with pulmonary edema, congestive hepatopathy or peripheral edema, catecholamine therapy, life-threatening cardiac arrhythmia, liver failure with an INR > 3.5 (Quick < 50%), a qSOFA score of ≥ 2 or acute renal failure with need of dialysis. The baseline data comprised patient characteristics, symptoms, co-morbidities, known microbiological colonization, preexisting medication, and laboratory and vital parameters.

We excluded patients with advanced COVID-19 stages at baseline. Furthermore, for the development of the algorithm and SACOV-19, we excluded patients with no documented information on laboratory or vital data (*n* = 279). Patients enrolled between 16 March and 14 July 2020 were included for the development of the method (discovery cohort). Patients enrolled between 15 July and 16 February 2021 were used for validation (validation cohort).

### Machine-learning and computation of SACOV-19

#### The workflow

**Fig. 1:**
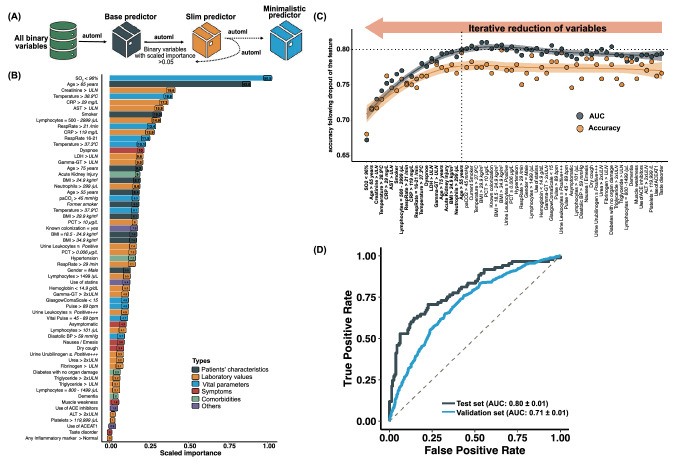
Machine-learning scheme and results. **A** Schematic workflow illustrating the iterative reduction of variables. First, the best performing predictor was selected based on all baseline variables (“base predictor”). Next, variables were removed following an iterative optimization procedure leading to the slim predictor and the minimalistic predictor. **B** Ranking of the variables for the “slim predictor” by their scaled importance. Values in parentheses depict the relative importance. **C** Performance (area under the curve [AUC] and accuracy) of predictors during the iterative optimization in a top down procedure. From right to left, the procedure started with *n* = 61 variables removing one variable at a time (displayed on the *x*-axis). The performance declined considerably at *n* = 21 variables which led to the selection of the minimalistic predictor just before this decline. **D** Receiver operating characteristics (ROC) curve of the minimalistic predictor on the test and validation set

All the aspects of data reporting, predictive modeling and validation reporting were performed in accordance with the TRIPOD guidelines [23]. To derive the machine learning based and the score based (SACOV-19) predictor, the following steps were performed (Fig. 1A):

1. Baseline data were preprocessed to calculate baseline variables (binary features).
2. The patient cohort of the discovery cohort was separated into a training and a test set.
3. Machine learning was performed based on all baseline variables and data of the training set yielding a predictor based on all variables (base predictor).
4. To improve robustness and interpretability, variables with low impact were iteratively removed. A predictor (“slim predictor”) with a reduced number of variables (*n* = 61) and a minimalistic predictor with *n* = 20 variables was obtained, the selection based on the performance on the test set.
5. SACOV-19 was developed by reducing the variables of the minimalistic predictor following a modified dynamic programming approach.
6. A browser-based web application of the minimalistic predictor and SACOV-19 was implemented.
7. SACOV-19 and the minimalistic predictor were evaluated using the data from the validation cohort.

### Identifying a predictor using the baseline variables

Using the data from the discovery cohort, patients were randomly separated into an endpoint-balanced training (80%) and a test set (20%). Endpoint balancing was achieved by stratification of the classes by inducing the sampling rate of patients progressing to advanced COVID-19 and reducing the sampling rate of patients not progressing to advanced COVID-19. Binary variables were defined for all baseline patient characteristics. To note, since in the LEOSS database also rational variables were given in categories, no information was lost by this binarization. Missing values or data documented as “unknown”, “not measured” or “not detected” were incorporated in the design of the binary variables. For details of the binary variable computation see Supplementary Text 1. These binary variables were used in the following data processing. The base predictor was constructed using the H2O.ai platform (https://www.h2o.ai) selecting automatically (with h2o.automl) the best suitable machine learning method on the training set. To save computational time, the selection of methods was limited to random forests, gradient boosting machines (gbm), extreme gradient boosting (XGBoost) and StackedEnsemble. The parameters of each method were optimized employing an internal tenfold cross-validation on the training set. The optimal method was then applied to the test set to assess the final performance. In each loop, the best performing predictor was identified from all obtained predictors using the performance measure logloss. The selection of predictors was based on the area under the curve (AUC > 0.75) and logloss < 0.50. A schematic representation of the procedure is shown in Supplementary Figure S1. Variables associated with the "base predictor" were selected according to their scaled importance above 0.05 to obtain the “slim predictor” which based on a reduced set of variables (*n* = 61). To obtain the best performing predictor based on a minimalistic set of variables, variables of the “slim predictor” were ranked according to their scaled importance. Of these, a smaller set of variables (*n* = 60) was selected by leaving out the lowest ranking variable, a new predictor trained on the training set and its performance evaluated on the test set. Again, the lowest ranking variable on the remaining set of variables was removed, a new predictor generated and tested in the same way. This procedure was repeated until no variable remained. Out of these predictors, the minimalistic predictor was selected showing the best tradeoff between good performance and minimal set of variables (see “Results”, XGBoost predictors). The robustness of the minimalistic predictor was evaluated by constructing supplementary (mutated) predictors leaving out one variable at a time. To estimate the robustness, the performance of these mutated predictors was compared to the performance of the minimalistic (wildtype) predictor. For the minimalistic predictor, a graphical user interface was implemented in R using the package Shiny and ggplot2. The computational core consists of functionalities employing the packages h2o and lime.

### Identifying discriminative single variables and the score (SACOV-19)

We estimated the discriminative power of each individual patient variable using the discovery set. The predictive power of each variable was estimated based on balanced accuracy. Patients with missing values for the tested variable were omitted. To identify the score (SACOV-19), we used the variables selected for the minimalistic predictor and combined up to a maximum of 16 variables into a predictive score. Each selected variable counted + 1. Together with a threshold T, the score predicted an advanced COVID-19 stage if at least T many of the (binary) variable values of the evaluated score equaled “yes” (+ 1) for a concrete patient. Varying the threshold from 0 to the length of the score, we computed the AUC for each score. We started with computing all scores of lengths two and stored the best 1000 of them according to their AUC. Next, the variables of each of these 1000 scores of lengths two were combined with one of the remaining variables. Doing this for all remaining variables yielded a list of scores of lengths three. Subsequently, we selected the 1000 best scores according to their AUC. This dynamic-programming-like procedure was repeated until a list of 1000 best scores of lengths 16 was compiled. Note, that this heuristic works in reasonable computational time. The rationale for this procedure was that we assumed that sub-scores of well performing scores also perform good. Indeed, we observed that bests-of-lists of length 200 (instead of 1000) yet comprised all the best scores. Out of the list of 16 best scores (with length 1–16), the optimal score was determined by selecting the score with the highest AUC on the test set of the 16 optimal scores. All data processing, modeling and assessment of performances was performed using R (version 3.6.3). Confidence intervals for the odds ratios were calculated using the package “fmsb_0.7.0” [24]. Further used packages were dplyr_1.0.5, h2o_3.30.0.7, lime_0.5.2, ggplot2_3.3.3, liqueueR_0.0.1, arsenal_3.6.2, caret_6.0-86, flexdashboard_0.5.2 and shiny_1.6.0.

## Results

### General characteristics of the study population

We included 3487 out of 6360 patients enrolled in LEOSS in our study, 1223/2819 patients for model discovery and 2264/3541 patients for validation (for details of the selection of patients, see “Methods” and Supplementary Figure S2). The analyzed patients were obtained from 117 LEOSS study sites located in Germany (94.8%, 3307/3487), Turkey (1.9% 66/3487), Belgium (0.8%, 29/3487), Switzerland (0.7%, 25/3487), the United Kingdom (0.7%, 25/3487), Latvia (0.7%, 24/3487), Spain (0.2%, 8/3487), Austria (0.06%, 2/3487), and Italy (0.03%, 1/3487). Patients were recruited either at university hospitals (60.6%, 2113/3487), community hospitals (36.5%, 1274/3487) or medical practices (2.8%, 100/3487). 91.5% of patients (3176/3470; 17 with missing information) were hospitalized during the observation period. In 74.1% of the patients (2345/3165; 322 with missing information) the first positive SARS-CoV-2 test (at baseline) was performed in an inpatient setting. 19.8% of the patients (582/2939; 548 with missing information) were documented as asymptomatic at baseline. Asymptomatic patients had predominantly more documented co-morbidities [21]. The clinical stage of 35.2% (1229/3487) patients worsened to the Advanced COVID-19 stage. The median days from the date of baseline to start of Advanced COVID-19 was five days (inter quartile range 2–7 days). An overview of the patient characteristics and clinical conditions at baseline of the validation cohort is given in Table 1. This data was kept untouched during machine learning and developing the SACOV-19 score. The patient characteristics of the discovery cohort are shown in Supplementary Table S1.

**Table 1:** Baseline characteristics of the validation cohort

	Patients which did not advance to the advanced COVID-19 stage	Patients which advanced to the advanced COVID-19 stage	Total	*p* value*
Included cases^a^	1463 (64.6%)	801 (35.4%)	2264	
Age				< 0.001**
< 26 years	132/1463 (9.0%)	19/801 (2.4%)	151/2264 (6.7%)	
26–45 years	417/1463 (28.5%)	89/801 (11.1%)	506/2264 (22.3%)	
46–65 years	479/1463 (32.7%)	289/801 (36.1%)	768/2264 (33.9%)	
> 65 years	435/1463 (29.7%)	404/801 (50.4%)	839/2264 (37.1%)	
Sex				< 0.001
Male	729/1463 (49.8%)	466/801 (58.2%)	1195/2264 (52.8%)	
Body mass index				< 0.001
< 18.5 kg/m^2^	34/779 (4.4%)	7/523 (1.3%)	41/1302 (3.1%)	
18.5–24.9 kg/m^2^	262/779 (33.6%)	154/523 (29.4%)	416/1302 (32.0%)	
25–29.9 kg/m^2^	274/779 (35.2%)	181/523 (34.6%)	455/1302 (34.9%)	
> 29.9 kg/m^2^	209/779 (26.8%)	181/523 (34.6%)	390/1302 (30.0%)	
Smoking status				0.034
Smoker or former smoker	158/572 (27.6%)	101/292 (34.6%)	259/864 (30.0%)	
Comorbidities				
Cardiovascular disease	565/1423 (39.7%)	496/791 (62.7%)	1061/2214 (47.9%)	< 0.001
Diabetes mellitus	202/1412 (14.3%)	178/781 (22.8%)	380/2193 (17.3%)	< 0.001
Pulmonary disease	129/1409 (9.2%)	137/782 (17.5%)	266/2191 (12.1%)	< 0.001
Hematological and/or oncological disease	122/1409 (8.7%)	119/775 (15.4%)	241/2183 (11.0%)	< 0.001
Neurological disease	237/1416 (16.7%)	185/782 (23.7%)	422/2198 (19.2%)	< 0.001
Kidney disease	141//1398 (10.1%)	165/762 (21.7%)	306/2160 (14.2%)	< 0.001
Other comorbidities^b^	99/1405 (7.0%)	77/779 (9.9%)	176/2184 (8.1%)	0.020
Body temperature				< 0.001
< 38.0 °C	913/1128 (80.9%)	286/404 (70.8%)	1199/2214 (78.3%)	
38.0—39.9 °C	210/1128 (18.6%)	107/404 (26.5%)	317/2214 (20.7%)	
> 39.9 °C	5/1128 (0.4%)	11/404 (2.7%)	16/2214 (1.0%)	
C-reactive protein				< 0.001
< 3 mg/L	228/899 (25.4%)	30/363 (8.3%)	258/1262 (20.4%)	
3–29 mg/L	417/899 (46.4%)	150/363 (41.3%)	567/1262 (44.9%)	
30–119 mg/L	204/899 (22.7%)	142/363 (39.1%)	346/1262 (27.4%)	
> 119 mg/L	50/899 (5.6%)	41/363 (11.3%)	91/1262 (7.2%)	

During the observational period. Patients with missing values for the respective variable were excluded in this statistic

*Using a *χ*^2^-test, **based on a multi-categorical *χ*^2^-test

^a^Age, body temperature and C-reactive protein are shown after binning categories of originally twelve, six and seven categories, respectively

^b^This included all other listed co-morbidities including connective tissue disease, peptic ulcer disease, chronic liver disease, liver cirrhosis, organ transplantation, rheumatic disease, HIV/AIDS

### Identifying a predictor based on a large set of baseline variables

**Fig. 2:**
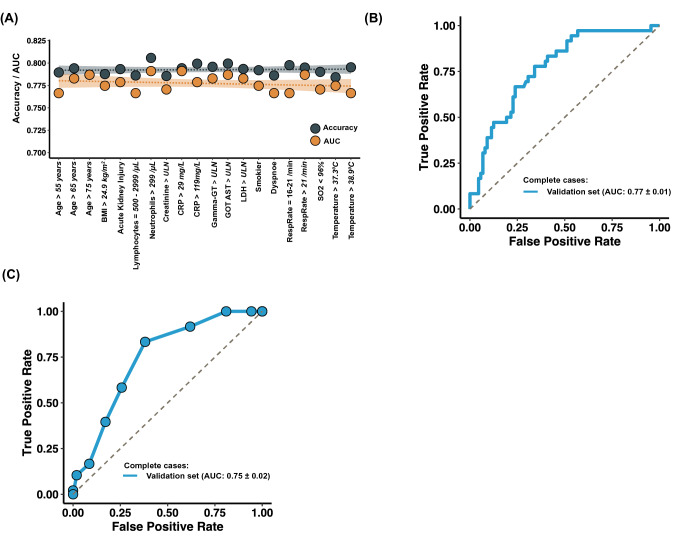
Stability and performance of the minimalistic predictor. **A** Performance of the minimalistic predictor when leaving out one variable at a time (displayed on the *x*-axis). Leaving out a single variable from the identified binary variables did not markedly influence the performance. **B** Receiver operating characteristics (ROC) curve of the minimalistic predictor on the validation set considering only patients without any missing value (in the list of *n* = 20 variables for the minimalistic predictor). **C** ROC curve of SACOV-19 based on patients of the validation set without missing values (in the list of the 11 patient variables of SACOV-19)

Our goal was to develop a predictor as the basis for deriving a score aiding the front-line physician identifying patients at risk developing Advanced COVID-19. We compiled 472 baseline patient variables (being present to the treating physician) as input for obtaining the “base predictor” and trained machines on data of the discovery cohort. Evaluating the performance on a test set (taken from the discovery cohort) (*n* = 244), the “base predictor” revealed decent performance (AUC = 0.79 ± 0.11, OR = 7.65 [95% CI 4.13–14.19]) (Supplementary Table S3). Next, we focused on a smaller set of variables for the prediction to simplify the interpretation and to improve generalizability. We obtained an optimized predictor based on *n* = 61 variables (slim predictor) showing an AUC of 0.80 ± 0.01, OR = 9.14 [95% CI 4.90–17.05] on the test set. Though the new predictor showed a similar performance as the base predictor, it consisted of a considerable reduced number of variables (Table S2, Fig. 1B). To further reduce the number of variables, we computed predictors by iteratively removing variables with minor importance leading to the minimalistic predictor with a similar performance (AUC = 0.80 ± 0.01, OR = 8.20 [95% CI 4.51–14.88], Table S2, Fig. 1C, Supplementary Figures S3A–B). The minimalistic predictor was based on the variables body-mass index (BMI > 24.9 kg/m^2^), smoking habit (smoker/former smoker), presence of acute kidney injury, dyspnea, oxygen saturation level (< 96%), body temperature (two thresholds, i.e. > 37.3 °C and > 38.9 °C), respiratory rate (two thresholds, > 16/min and > 21/min), C-reactive protein (CRP, 2 thresholds, > 29 and > 119 mg/L), creatinine (≥ ULN, upper limit of normal), LDH (≥ ULN), AST (≥ ULN), gamma-GT (≥ ULN), lymphocyte counts (≥ 3000/µL), and neutrophil counts (≥ 3000/µL). Age was employed along three different thresholds (> 55, > 65 and > 75 years) reflecting the continuously increasing risk with increasing age. Using the (unseen) data from the validation cohort, the minimalistic predictor showed an AUC = 0.71, OR = 4.41 [95% CI 3.57–5.46]. Receiver operating characteristics (ROC) curve of the minimalistic predictor are shown in Fig. 1D. Further performance values are shown in Table S1. A predictor is estimated to be robust if it performs similar under varying input conditions [25]. We constructed predictors by randomly dropping single variables. We observed that this did not influence the performance **(**Fig. 2A**)**, reflecting the robustness of the minimalistic predictor. Hitherto, the results were based on patients containing missing values. To assess the impact of missing values on the predictive power, we applied the minimalistic predictor to data of patients without missing values for any of the 20 patient variables. We observed a slightly better prediction performance (on the validation set AUC = 0.77 ± 0.02, OR = 6.78 [95% CI 2.74–16.65] and balanced accuracy: 0.72 ± 0.01 using *n* = 124 patients, Table S3, Fig. 2B, Supplementary Figures S3C).

To summarize, we constructed and internally validated a minimalistic predictor based on 20 patient variables comprising patient characteristics such as age and body mass index, but also vital parameters such as body temperature, respiration and lung parameters, several blood laboratory parameters such as CRP, LDH and creatinine levels, and acute kidney injury at diagnosis. The predictor showed good and stable performance in predicting the development to the advanced COVID-19 stage.

### Identifying a predictive score and the discriminative power of single variables

For clinical implementation, we developed an early warning score. Starting with the 20 variables from the minimalistic predictor, we applied the score optimization procedure (described in “Methods”) and identified a predictive score (SACOV-19) based on 11 patient characteristics or 14 binary variables including three binary variables originating from the same categorical variables. The performance was similar as for the machine learning-based predictors (AUC 0.80 ± 0.01) for the discovery set. For the validation set, the AUC was 0.73 ± 0.01. The composition of SACOV-19 is shown in Table 2. A high sensitivity is of particular clinical relevance reducing misclassification of patients in need of hospitalization and close monitoring and who possibly could benefit for medical treatment. This can be achieved using lower thresholds. Selecting a threshold of four, we obtained a sensitivity of 0.90 and an absolute risk reduction of ARR = 0.34 for the validation cohort. 45.7% (717/1,570) of patients with a score of at least 4 deteriorated to advanced COVID-19.

**Table 2:** Variables of SACOV-19

Variables	Range/value	Score value
Age	66–75 years	+ 1
	> 75 years	+ 2
Body mass index	> 24.9 kg/${\mathrm{m}}^{2}$	+ 1
Smoker	Smoker or former smoker^a^	+ 1
Respiratory rate	> 21 per min^a^	+ 1
Oxygen saturation	< 96%	+ 1
Temperature	37.4 °C–38.9 °C^a^	+ 1
	> 38.9 °C	+ 2
CRP	30–119 mg/L^a^	+ 1
	> 119 mg/L	+ 2
LDH	Above normal	+ 1
Lymphocyte counts	< 500/µL or > 2999/µL	+ 1
Acute kidney injury^b^	Yes	+ 1
Dyspnea	Yes	+ 1

^a^Or unknown

^b^Acute kidney injury was defined based on the diagnosis of the first line physician

Removing patients with a missing value in at least one of the 14 binary variables, improved the performance for the discovery (AUC = 0.83 ± 0.02, *n* = 120 patients) and the validation set (AUC = 0.75 ± 0.02, *n* = 153 patients) (Fig. 2C, Supplementary Figures S3D). Table 3 shows the performances for three different thresholds. To test if our score only works within a hospital setting, we computed the performance also for outpatients and asymptomatic patients. For outpatients (*n* = 28, after removal of patients with at least one NA in the score variables) the sensitivity was 82% and specificity 53%. For the asymptotic patients the sensitivity was 67% with a specificity of 81% (threshold = 4, *n* = 29 after removal of patients with at least one NA in the score variables). However, both results show only the tendency as their lower confidence values were not above one, assumedly due to the low patient numbers. To evaluate the predictive power of single variables, we computed their individual performance as a predictor to develop an advanced COVID-19 stage. Table 3 shows the results. The best single variable was oxygen saturation (SO2) smaller than 96% with an AUC of 0.63 ± 0.01 (OR = 3.07 [95% CI 2.34–4.04]). Notably, the top five discriminating variables (oxygen saturation, age, CRP, LDH and temperature) are all part of the minimalistic predictor *and* of SACOV-19 showing the consistency of the results and the principal relevance of these five variables.

**Table 3:** Performance of SACOV-19 and single variables removing patients with missing values

	Balanced accuracy	Sensitivity	Specificity	Odds ratio	95% CI	
					Lower	Upper
Performance of the score at different thresholds for the validation set						
SACOV-19 ≥ 5	0.66	0.58	0.74	4.04	1.97	8.32
SACOV-19 ≥ 4	0.73	0.83	0.62	8.13	3.45	19.11
SACOV-19 ≥ 3	0.65	0.92	0.38	6.77	2.26	20.27
Performance of single binary variables						
Oxygen saturation < 96%^a^	**0.63**	**0.53**	**0.73**	**3.07**	**2.34**	**4.04**
Age > 65 years	**0.62**	**0.60**	**0.65**	**2.75**	**2.16**	**3.51**
CRP > 29 mg/L	**0.62**	**0.53**	**0.70**	**2.67**	**2.04**	**3.49**
LDH > ULN	**0.61**	**0.65**	**0.57**	**2.40**	**1.80**	**3.21**
Temperature > 37.3 °C	**0.60**	**0.68**	**0.53**	**2.37**	**1.83**	**3.08**
Urine total protein positive	0.60	0.57	0.63	2.27	1.44	3.60
Hypertension	0.59	0.54	0.64	2.13	1.68	2.72
IL6 > 199 ng/L	0.59	0.29	0.90	3.48	1.88	6.45
Lymphocyte counts < 800/µL	0.59	0.47	0.71	2.17	1.59	2.94
D-dimer > ULN	0.59	0.70	0.47	2.08	1.41	3.08
Creatinine > ULN ^b^	*0.58*	*0.34*	*0.81*	*2.22*	*1.64*	*2.99*
BMI > 24.9 kg/${\mathrm{m}}^{2}$	**0.57**	**0.64**	**0.51**	**1.84**	**1.34**	**2.53**
Urea > ULN	0.57	0.29	0.86	2.40	1.70	3.40
Ferritin > 299 ng/mL	0.57	0.65	0.49	1.77	1.15	2.72
Urine ketone bodies positive	0.57	0.32	0.82	2.10	1.26	3.49
PaO_2_ < 80 mmHg	0.57	0.46	0.67	1.76	0.92	3.35
AST > ULN	*0.56*	*0.35*	*0.78*	*1.89*	*1.37*	*2.59*
Respiratory rate > 21 per min	**0.56**	**0.29**	**0.84**	**2.09**	**1.43**	**3.05**
Use of ACE/AT1	0.56	0.40	0.72	1.74	1.36	2.25
Hemoglobin < 12 g/dL	0.56	0.37	0.75	1.78	1.34	2.35

*ULN* upper limit of normal 6/29/2021 10:54:00 AM

^a^Variables highlighted in bold are part of the minimal predictor and SACOV-19

^b^Variables highlighted in italic are part of the minimal predictor but not of SACOV-19

In summary, using the preselected variables from the minimalistic predictor enabled to define a clinical score comprising eleven patient variables with a good performance which is comparable to the machine learning-based predictors.

### Implementation of the machine learning-based predictor into a web interface

To illustrate the performance of the minimalistic predictor, we designed a graphical user interface for a quick entry of the values of potential patient variables, followed by the prediction of the investigated endpoint**.** The web interface (http://www.klinikum.uni-muenchen.de/Medizinische-Klinik-und-Poliklinik-II/de/sacov19app/index.html, login: user, password: sacov19) provides the user with the model-based estimated probability of the patient to develop an advanced COVID-19 stage, the odds ratio, SACOV-19 and the model prediction. Moreover, it provides several graphical presentations to illustrate the impact of the specific variables on the decision. Supplementary Figure S4 and movie M1 shows the web front-end and illustrates its usage (for scientific use).

## Discussion

We computed and validated a predictor and associated predictive score (SACOV-19) to predict a complicated or more severe COVID-19 stage in patients, who were tested positive for SARS-CoV-2 and presented at mainly inpatient settings asymptomatic or with mild COVID-19 symptoms. SACOV-19 is based on standard parameters, which can be acquired in most hospital and out-patient settings. In addition, we implemented a browser-based interactive graphical user interface making the data-driven model accessible to the research community.

Though most patients presenting asymptomatic or with mild COVID-19 symptoms do not require medical treatment, some patients rapidly deteriorate and need medical intervention [17, 26]. By focusing on complicated or more severe COVID-19 as the endpoint, our score (SACOV-19) identifies patients requiring medical intervention and hospitalization. For asymptomatic/mild COVID-19 patients with increased risk predicted by our score, the attending physician might consider hospitalization or close follow-up. A high-risk result might also enter risk–benefit considerations when evaluating medical treatments with possible side effects. In turn, supporting the decision to discharge an asymptomatic/mild COVID-19 patient according to our score, enables physicians to prioritize patients in need for hospitalization and close monitoring.

As of now, management decisions for asymptomatic/mild COVID-19 patient are mainly based on the presence of risk factors, the clinical judgment of the attending physicians and the available resources [17]. Unfortunately, course and outcome of COVID-19 are heterogeneous complicating this situation. Risk factors such as higher age, high BMI, male sex or arterial hypertension have been associated with poorer prognosis. However, they are also highly prevalent in patients with mild or asymptomatic courses [5]. Earlier studies evaluated general disease severity scores such as CRB65, NEWS2, or qSOFA in COVID-19. Mostly, these scores were validated for risk of progression to severe COVID-19 or death, to guide IMC/ICU admission in hospitalized patients [27–30]. Notably, patients of our cohort showed a very indistinctive qSOFA score at baseline, indicating its unsuitability for identifying asymptomatic patients or with mild COVID-19 who are at risk of developing an advanced stage (58% accuracy for a threshold of one, and Glasgow Coma Scale ≤ 12 instead of 14). Scores specifically developed for risk of progression in COVID-19 like the COVID-GRAM, Brescia-COVID Respiratory Severity Scale (BCRSS) or 4C Mortality Score most entirely focus on the progression to severe respiratory impairment and death not taking the early risk of progression into a complicated stage into consideration [6, 8, 12, 31]. Exceptions are the CALL and EWAS score and the score published by Huang* et al.* [32], which were designed to predict risk for progression to advanced COVID-19. However, these scores were based on a relatively small patient cohort [32, 33]. Though in validation studies, their performance in predicting the progression to complicated or more severe COVID-19 was poor (AUC < 0.67) [13, 34]. To note, we could not evaluate these scores and most of the published scores for the critical endpoint as the needed thresholds for calculating the according variables are more complex and were not collected in LEOSS. LEOSS data were collected using predefined categories to preserve the anonymous data collection protocol. In the 4C Mortality score [8], for example, which was rated as high quality [12], categories for age, respiratory rate, oxygen saturation, urea and C reactive protein were not mappable to LEOSS. In future research the 4C mortality score, for example, could be adapted to the LEOSS data and could be evaluated on advanced COVID-19.

SACOV-19 is based on eleven patient characteristics (14 binary variables) which are often documented at first presentation. In line with previous studies, SACOV-19 shows that patients of higher age, higher BMI, and smokers or former smokers have a higher risk for advanced COVID-19 courses [5, 12, 13, 26]. The respiratory parameters oxygen saturation, respiratory rate and feeling of dyspnea are included in SACOV-19 emphasizing the importance of examining pulmonary parameters at initial presentation.

A strength of the study is that it is based on data of a well-documented and curated multinational COVID-19 registry supported by the German Center for Infection Research and German Infectious Disease Society, and a well set up machine learning procedure. We trained the SACOV-19 on a discovery cohort including only patients from the first wave of the COVID-19 pandemic. SACOV-19 was tested on an independent validation cohort comprising patients from the first to the third wave, which have been collected after the development of the score. COVID-19 is a newly emerging infectious disease, for which the knowledge and standard of care evolved. Hence one may argue that our score which was developed based on data from March to July 2020 may not be useful anymore. But, most treatment options to date are administered after a COVID-19 disease deterioration [35] which is our endpoint and hence would not affect the predictiveness of our score. Indeed, when we tested SACOV-19 on an independent validation cohort comprising patients from the first to the third wave (in which potential changes of care may have occurred), we didn’t recognize a drop in performance. The SACOV-19 stands out because it has been evaluated across regions and sectors. At the time of manuscript preparation, it contained, to our knowledge, the largest German data collection of comprehensive clinical data on high-risk patients. [36]. Nevertheless, until now, the investigated patients may limit its general applicability. Most of the patients received care in an inpatient setting. When testing our score on outpatients we observed a similar performance result, however, we had only *n* = 28 outpatients for this analysis and could hence not get a significant result. Furthermore, the majority of patients exhibited a mild disease and did not advance to the complicated phase. Therefore, patients with co-morbidities could have been overrepresented in our cohort, as these patients were mainly admitted without severe symptoms [21]. To show the general applicability of our score, a further, clinical trial is necessary. We actually plan a trial testing in a primary care setting if SACOV-19 acceptably predicts COVID-19 deterioration.

While we included a large cohort of patients, a limitation is that the majority of patients were included at German health care facilities. Our results may not be fully applicable to countries or regions with different demographics or resource settings. Most of the patients received care in an inpatient setting. The majority exhibited a mild disease and did not advance to the complicated phase. Therefore, patients with co-morbidities could be overrepresented in our cohort, as these patients were mainly admitted without severe symptoms [21]. Another caveat may be the high number of missing values for specific variables and, in particular, some laboratory values, as not all parameters were collected at the day of the first positive SARS-CoV-2 test. For example interleukin 6 has been shown to have predictive power for a severe COVID-19 course [37] but was not selected by our algorithms, possibly due to its high number of missing values. Furthermore, thresholds for parameters were predefined in the study protocol. Metric available data could improve prediction models. The web application was designed for research use making our predictor accessible to the research community.

## Conclusion

We present a robust machine learning-based predictor and, from this, a score (SACOV-19) to identify patients with predominantly known risk factors at risk of developing an advanced COVID-19 stage. To make it accessible to the research community, the predictor is available through a web interface. The predictor and score encompass patient variables which are commonly assessed in the primary care setting and are easily available. SACOV-19 may promote clinical decision making when it is essential assessing the risk for complicated or more advanced COVID-19 stages. Prospective clinical studies are needed to prove its reliability, particularly in countries or regions with different demographics or resource settings.

## Supplementary Information

Below is the link to the electronic supplementary material.

Supplementary file1 (DOCX 6699 kb)

Supplementary file2 (MOV 6187 kb)

## References

[1] Okada P, Buathong R, Phuygun S, Thanadachakul T, Parnmen S, Wongboot W, et al. Early transmission patterns of coronavirus disease 2019 (COVID-19) in travellers from Wuhan to Thailand, January 2020. Eurosurveillance 2020;25:2000097. 10.2807/1560-7917.ES.2020.25.8.2000097 PMC7055038 32127124

[2] Coronaviridae Study Group of the International Committee on Taxonomy of Viruses (2020). The species severe acute respiratory syndrome-related coronavirus: classifying 2019-nCoV and naming it SARS-CoV-2. Nat Microbiol.

[3] WHO. Weekly operational update on COVID-19. 2020. http://www.who.int.

[4] Guan W, Ni Z, Hu Y, Liang W, Ou C, He J (2020). Clinical characteristics of coronavirus disease 2019 in China. N Engl J Med.

[5] Zhou F, Yu T, Du R, Fan G, Liu Y, Liu Z (2020). Clinical course and risk factors for mortality of adult inpatients with COVID-19 in Wuhan, China: a retrospective cohort study. Lancet.

[6] Liang W, Liang H, Ou L, Chen B, Chen A, Li C (2020). Development and validation of a clinical risk score to predict the occurrence of critical illness in hospitalized patients with COVID-19. JAMA Intern Med.

[7] Clift AK, Coupland CAC, Keogh RH, Diaz-Ordaz K, Williamson E, Harrison EM, et al. Living risk prediction algorithm (QCOVID) for risk of hospital admission and mortality from coronavirus 19 in adults: national derivation and validation cohort study. BMJ. 2020;371:m3731. 10.1136/bmj.m3731 PMC7574532 33082154

[8] Knight SR, Ho A, Pius R, Buchan I, Carson G, Drake TM, et al. Risk stratification of patients admitted to hospital with covid-19 using the ISARIC WHO Clinical Characterisation Protocol: development and validation of the 4C Mortality Score. BMJ. 2020;370:m3339. 10.1136/bmj.m3339 PMC7116472 32907855

[9] Carr E, Bendayan R, Bean D, Stammers M, Wang W, Zhang H (2021). Evaluation and improvement of the National Early Warning Score (NEWS2) for COVID-19: a multi-hospital study. BMC Med.

[10] Liu S, Yao N, Qiu Y, He C (2020). Predictive performance of SOFA and qSOFA for in-hospital mortality in severe novel coronavirus disease. Am J Emerg Med.

[11] Chang MC, Park Y-K, Kim B-O, Park D (2020). Risk factors for disease progression in COVID-19 patients. BMC Infect Dis.

[12] Wynants L, Van Calster B, Collins GS, Riley RD, Heinze G, Schuit E, et al. Prediction models for diagnosis and prognosis of covid-19: systematic review and critical appraisal. BMJ. 2020;369:m1328. 10.1136/bmj.m1328 PMC7222643 32265220

[13] Gupta RK, Marks M, Samuels THA, Luintel A, Rampling T, Chowdhury H (2020). Systematic evaluation and external validation of 22 prognostic models among hospitalised adults with COVID-19: an observational cohort study. Eur Respir J.

[14] Gandhi RT, Lynch JB, Del Rio C (2020). Mild or moderate Covid-19. N Engl J Med.

[15] Wu Z, McGoogan JM (2020). Characteristics of and important lessons from the coronavirus disease 2019 (COVID-19) outbreak in China: summary of a report of 72 314 cases from the Chinese Center for Disease Control and Prevention. JAMA.

[16] Attaway AH, Scheraga RG, Bhimraj A, Biehl M, Hatipoğlu U (2021). Severe covid-19 pneumonia: pathogenesis and clinical management. BMJ.

[17] Alhazzani W, Møller MH, Arabi YM, Loeb M, Gong MN, Fan E (2020). Surviving Sepsis Campaign: guidelines on the management of critically ill adults with coronavirus disease 2019 (COVID-19). Intensive Care Med.

[18] Darley DR, Dore GJ, Cysique L, Wilhelm KA, Andresen D, Tonga K (2021). Persistent symptoms up to four months after community and hospital-managed SARS-CoV-2 infection. Med J Aust.

[19] Carfì A, Bernabei R, Landi F, Gemelli Against COVID-19 Post-Acute Care Study Group (2020). Persistent symptoms in patients after acute COVID-19. JAMA.

[20] Weerahandi H, Hochman KA, Simon E, Blaum C, Chodosh J, Duan E (2021). Post-discharge health status and symptoms in patients with severe COVID-19. J Gen Intern Med.

[21] Jakob CEM, Borgmann S, Duygu F, Behrends U, Hower M, Merle U (2020). First results of the “Lean European Open Survey on SARS-CoV-2-Infected Patients (LEOSS)”. Infection.

[22] Jakob CEM, Kohlmayer F, Meurers T, Vehreschild JJ, Prasser F (2020). Design and evaluation of a data anonymization pipeline to promote Open Science on COVID-19. Sci Data.

[23] Collins GS, Reitsma JB, Altman DG, Moons K (2015). Transparent reporting of a multivariable prediction model for individual prognosis or diagnosis (TRIPOD): the TRIPOD statement. BMC Med.

[24] Nakazawa M. Package ‘fmsb’, Functions for medical statistics book with some demographic data, Version 0.7.1. CRAN Repository. 2021. https://cran.r-project.org.

[25] Menon V, Larson K. Algorithmic stability in fair allocation of indivisible goods among two agents. 2020. arXiv: 2007.15203.

[26] Zheng Z, Peng F, Xu B, Zhao J, Liu H, Peng J (2020). Risk factors of critical and mortal COVID-19 cases: a systematic literature review and meta-analysis. J Infect.

[27] Fan G, Tu C, Zhou F, Liu Z, Wang Y, Song B (2020). Comparison of severity scores for COVID-19 patients with pneumonia: a retrospective study. Eur Respir J.

[28] Gidari A, De Socio GV, Sabbatini S, Francisci D (2020). Predictive value of National Early Warning Score 2 (NEWS2) for intensive care unit admission in patients with SARS-CoV-2 infection. Infect Dis.

[29] Ihle-Hansen H, Berge T, Tveita A, Rønning EJ, Ernø PE, Andersen EL, Wang CH, Tveit A, Myrstad M. COVID-19: Symptoms, course of illness and use of clinical scoring systems for the first 42 patients admitted to a Norwegian local hospital. Tidsskr Nor Laegeforen. 2020;140(7). 10.4045/tidsskr.20.0301 32378844

[30] Smith GB, Redfern OC, Pimentel MA, Gerry S, Collins GS, Malycha J (2019). The National Early Warning Score 2 (NEWS2). Clin Med.

[31] Duca A, Piva S, Focà E, Latronico N, Rizzi M (2020). Calculated decisions: Brescia-COVID Respiratory Severity Scale (BCRSS)/Algorithm. Emerg Med Pract.

[32] Huang H, Cai S, Li Y, Li Y, Fan Y, Li L (2020). Prognostic Factors for COVID-19 pneumonia progression to severe symptoms based on earlier clinical features: a retrospective analysis. Front Med (Lausanne)..

[33] Guo Y, Liu Y, Lu J, Fan R, Zhang F, Yin X, et al. Development and validation of an early warning score (EWAS) for predicting clinical deterioration in patients with coronavirus disease 2019. medRxiv preprint, 10.1101/2020.04.17.20064691.

[34] Grifoni E, Valoriani A, Cei F, Vannucchi V, Moroni F, Pelagatti L, et al. The CALL score for predicting outcomes in patients with COVID-19. Clin Infect Dis. 2021;72:182–183. 10.1093/cid/ciaa686 PMC7314186 32474605

[35] Lamontagne F, Agoritsas T, Siemieniuk R, Rochwerg B, Bartoszko J, Askie L, et al. A living WHO guideline on drugs to prevent covid-19. BMJ. 2021;372:n526. 10.1136/bmj.n526 33649077

[36] Moons KGM, Altman DG, Vergouwe Y, Royston P (2009). Prognosis and prognostic research: application and impact of prognostic models in clinical practice. BMJ.

[37] Herold T, Jurinovic V, Arnreich C, Lipworth BJ, Hellmuth JC, von Bergwelt-Baildon M (2020). Elevated levels of IL-6 and CRP predict the need for mechanical ventilation in COVID-19. J Allergy Clin Immunol.

