# Supplementary material for: Prediction of COVID-19 deterioration in high-risk patients at diagnosis: an early warning score for advanced COVID-19 developed by machine learning
Source: Infection. 2021 Jul 19;50(2):359–70. doi: 10.1007/s15010-021-01656-z (PMC8287547; doi:10.1007/s15010-021-01656-z)
Supplement: Supplementary file 1 — Supplementary file1 (DOCX 6699 kb) [file 15010_2021_1656_MOESM1_ESM.docx]

**Supplementary material**

**Supplementary Text S1**

**Preprocessing of the patient variables**

Baseline variables with more than 90% missing values were excluded. A missing value was defined for either missing entries in the database or entries with the value “unknown”, “not measured” or “not detected”. As the machine learning procedure based on decision trees, we binarized all variables. Nominal binary variables (e.g. gender) remained unchanged. For ordinal variables (e.g. age, categorical, can be ordered), a threshold between each pair of consecutive categories was defined. For each threshold, a binary variable was defined separating the patients into smaller and higher than the threshold. In addition, for ordinal variables, u-shaped (binary) variables were computed separating the patients into “normal” and “not normal” (either more/higher than normal or less/lower than normal). The u-shaped variables are shown in Table S3. Altogether, we assembled n=236 binary variables. To cope for patients with a missing value in a certain variable, we doubled all variables and assigned patients with a missing value in a certain variable to each value (Yes/No) of the variable, respectively (missing value equaled the “Yes” category in the first replicate of the variable and the “No” category in the second replicate of the variable). By this we finally assembled n=472 variables.

**Supplementary Tables**

**Table S1.** Baseline characteristics of the discovery cohort*

|  | Asymptomatic or mild symptoms** | Advanced COVID-19 stage | Total | P-value*** |
| --- | --- | --- | --- | --- |
| Included cases**** | 795 (65.0%) | 428 (35.0%) | 1,223 |  |
| Age |  |  |  | <0.001***** |
| < 26 years | 55/795 (6.9%) | 15/428 (3.5%) | 70/1,223 (5.7%) |  |
| 26 - 45 years | 175/795 (22.0%) | 43/428 (10.0%) | 218/1,223 (17.8%) |  |
| 46 - 65 years | 295/795 (37.1%) | 117/428 (27.3%) | 412/1,223 (33.7%) |  |
| > 65 years | 270/795 (34.0%) | 253/428 (59.1%) | 523/1,223 (42.8%) |  |
| Sex |  |  |  | 0.020 |
| Male | 424/795 (53.3%) | 258/428 (60.3%) | 682/1,223 (55.8%) |  |
| Body Mass Index |  |  |  | 0.004 |
| < 18.5 kg/m² | 20/460 (4.3%) | 9/243 (3.7%) | 29/703 (4.2%) |  |
| 18.5 – 24.9 kg/m² | 213/460 (46.3%) | 78/243 (32.1%) | 291/702 (41.4%) |  |
| 25 – 29.9 kg/m² | 146/460 (31.7%) | 92/243 (37.9%) | 238/703 (33.9%) |  |
| > 29.9 kg/m² | 91/460 (17.6%) | 64/243 (26.3%) | 145/703 (20.6%) |  |
| Smoking status |  |  |  | 0.022 |
| Smoker or former smoker | 96/433 (22.2%) | 61/208 (29.3%) | 157/641 (24.5%) |  |
| Comorbidities |  |  |  |  |
| Cardiovascular disease | 334/771 (43.3%) | 266/422 (63.0%) | 600/1,193 (50.3%) | <0.001 |
| Diabetes mellitus | 92/779 (11.8%) | 105/417 (24.9%) | 197/1,196 (16.5%) | <0.001 |
| Pulmonary disease | 86/764 (11.3%) | 64/403 (15.9%) | 150/1,167 (12.9%) | 0.151 |
| Hematological and/or oncological disease | 112/778 (14.4%) | 78/403 (19.4%) | 190/1,181 (16.1%) | 0.118 |
| Neurological disease | 134/714 (18.8%) | 113/403 (28.0%) | 247/1,117 (22.1%) | <0.001 |
| Kidney disease | 81/780 (10.4%) | 78/416 (18.8%) | 159/1,196 (13.3%) | <0.001 |
| Other comorbidities****** | 84/763 (11.0%) | 65/401 (16.2%) | 149/1,164 (12.8%) | 0.013 |
| Body temperature |  |  |  | <0.001 |
| < 38.0°C | 512/702 (72.9%) | 203/380 (53.4%) | 715/1,082 (66.1%) |  |
| 38.0 - 39.9°C | 181/702 (25.8%) | 169/380 (44.5%) | 350/1,082 (32.3%) |  |
| > 39.9°C | 9/702 (1.3%) | 8/380 (2.1%) | 17/1,082 (1.6%) |  |
| C-reactive protein |  |  |  | < 0.001 |
| < 3 mg/L | 182/684 (26.6%) | 46/337 (13.6%) | 228/1,021 (22.3%) |  |
| 3- 29 mg/L | 298/684 (43.6%) | 112/337 (33.2%) | 410/1,021 (40.2%) |  |
| 30 - 119 mg/L | 173/684 (25.3%) | 128/337 (37.9%) | 301/1,021 (29.5%) |  |
| >119 mg/L | 31/684 (4.5%) | 51/337 (15.1%) | 82/1,021 (8.0%) |  |

* during the observational period. Patients with missing values for the respective variable were excluded in this statistic.

** Patients which were asymptomatic or with symptoms of the upper respiratory tract, fever, nausea, emesis or diarrhea.

*** using a χ²-test

****Age, body temperature and C-reactive protein are shown after binning categories of originally twelve, six and seven categories, respectively.

***** based on a multi-categorical χ²-test

****** This included all other listed comorbidities including connective tissue disease, peptic ulcer disease, chronic liver disease, liver cirrhosis, organ transplantation, rheumatic disease, HIV/AIDS

**Table S2.** Performance of the identified predictors

|  | **Discovery set**  **(n= 979)** | | | **Test set**  (n = 244) | | | **Validation Set (n = 2264)** |
| --- | --- | --- | --- | --- | --- | --- | --- |
| ***Predictors*** | Base | Slim | Minimalistic | Base | Slim | Minimalistic | Minimalistic |
| ***Number of variables*** | 236 | 61 | 20 | 236 | 61 | 20 | 20 |
| ***AUC*** | 0.90 ± 0.01 | 0.89 ± 0.01 | 0.84 ± 0.01 | 0.79 ± 0.01 | 0.79 ± 0.01 | 0.80 ± 0.01 | 0.71 ± 0.01 |
| ***Logloss**** | 0.40 | 0.50 | 0.46 | 0.51 | 0.52 | 0.57 | 0.60 |
| ***Gini***** | 0.80 | 0.78 | 0.69 | 0.58 | 0.59 | 0.59 | 0.41 |
| ***MPCE****** | 0.16 | 0.17 | 0.22 | 0.26 | 0.25 | 0.25 | 0.34 |

*

*Logloss evaluates how close a predicted value (uncalibrated probability estimate) is to the actual target value.

**Gini quantifies the inequality among values of a frequency distribution

***MPCE: Mean Per-Class Error is the average of the errors of each class

**Table S3:** Results of the minimalistic predictor on patients without any missing data.

| **Predictor** | **Number of variables** | **Test set**  **(n = 74)** | | | | **Validation set**  **(n = 124)** | | | |
| --- | --- | --- | --- | --- | --- | --- | --- | --- | --- |
|  |  | *AUC* | *Logloss^*^* | *Gini^**^* | *MPCE **** | *AUC* | *Logloss^*^* | *Gini^**^* | *MPCE **** |
| Minimalistic | 20 | 0.89 ± 0.01 | 0.40 ± 0.02 | 0.77 ± 0.03 | 0.18 ± 0.01 | 0.77 ± 0.01 | 0.51 ± 0.02 | 0.54 ± 0.02 | 0.28 ± 0.02 |

*Logloss evaluates how close a predicted value (uncalibrated probability estimate) is to the actual target value.

**Gini quantifies the inequality among values of a frequency distribution

***MPCE: Mean Per-Class Error is the average of the errors of each class

**Table S4:** U-shaped binary variables and their definition.

| **Binary variable** | **Definition** |
| --- | --- |
| BMI normal | 18.5 – 24.9 kg/m² |
| BMI slightly abnormal | <18.5 kg/m² or 25.0 – 29.9 kg/m² |
| BMI abnormal | 30.0 – 34.9 kg/m² |
| Leukocytes normal | 4,000 – 11,999 /µL |
| Leukocytes slightly abnormal | 1,000 – 3,999 /µL or 12,000 – 15,999 /µL |
| Leukocytes abnormal | < 1,000 /µL or 16,000 – 19,999 /µL |
| Leukocytes strongly abnormal | ≥ 20,000 /µL |
| Lymphocytes normal | 800 - 1499 /µL |
| Lymphocytes slightly abnormal | 500 - 799 /µL or 1,500 - 2,999 /µL |
| Lymphocytes abnormal | 300 - 499 /µL or ≥ 3,000 /µL |
| Lymphocytes strongly abnormal | 100 - 299 /µL |
| Lymphocytes severely abnormal | < 100 /µL |
| Neutrophils normal | 2,000 - 8,999 /µL |
| Neutrophils slightly abnormal | 500 - 1,999 /µL or ≥ 9,000 /µL |
| Neutrophils abnormal | 300 - 499 /µL |
| Neutrophils strongly abnormal | 100 - 299 /µL |
| Neutrophils severely abnormal | < 100 /µL |
| Platelets normal | 120,000 - 449,999 /µL |
| Platelets slightly abnormal | 50,000 - 119,999 /µL or 450,000 - 799,000 /µL |
| Platelets abnormal | 10,000 - 49,999 /µL or 800,000 - 1,199,999 /µL |
| Platelets strongly abnormal | < 10,000 /µL or ≥ 1,200,000 /µL |
| Systolic blood pressure normal | 100 - 139 mmHg |
| Systolic blood pressure slightly abnormal | 80 - 99 mmHg or 140 - 179 mmHg |
| Systolic blood pressure abnormal | < 80 mmHg or ≥ 180 mmHg |
| Diastolic blood pressure normal | 60 - 89 mmHg |
| Diastolic blood pressure slightly abnormal | 40 - 59 mmHg or 90 - 109 mmHg |
| Diastolic blood pressure abnormal | < 40 mmHg or ≥ 110 mmHg |
| Pulse normal | 45 - 89 bpm |
| Pulse slightly abnormal | < 45 bpm or 90 - 119 bpm |
| Pulse abnormal | > 119 bpm |


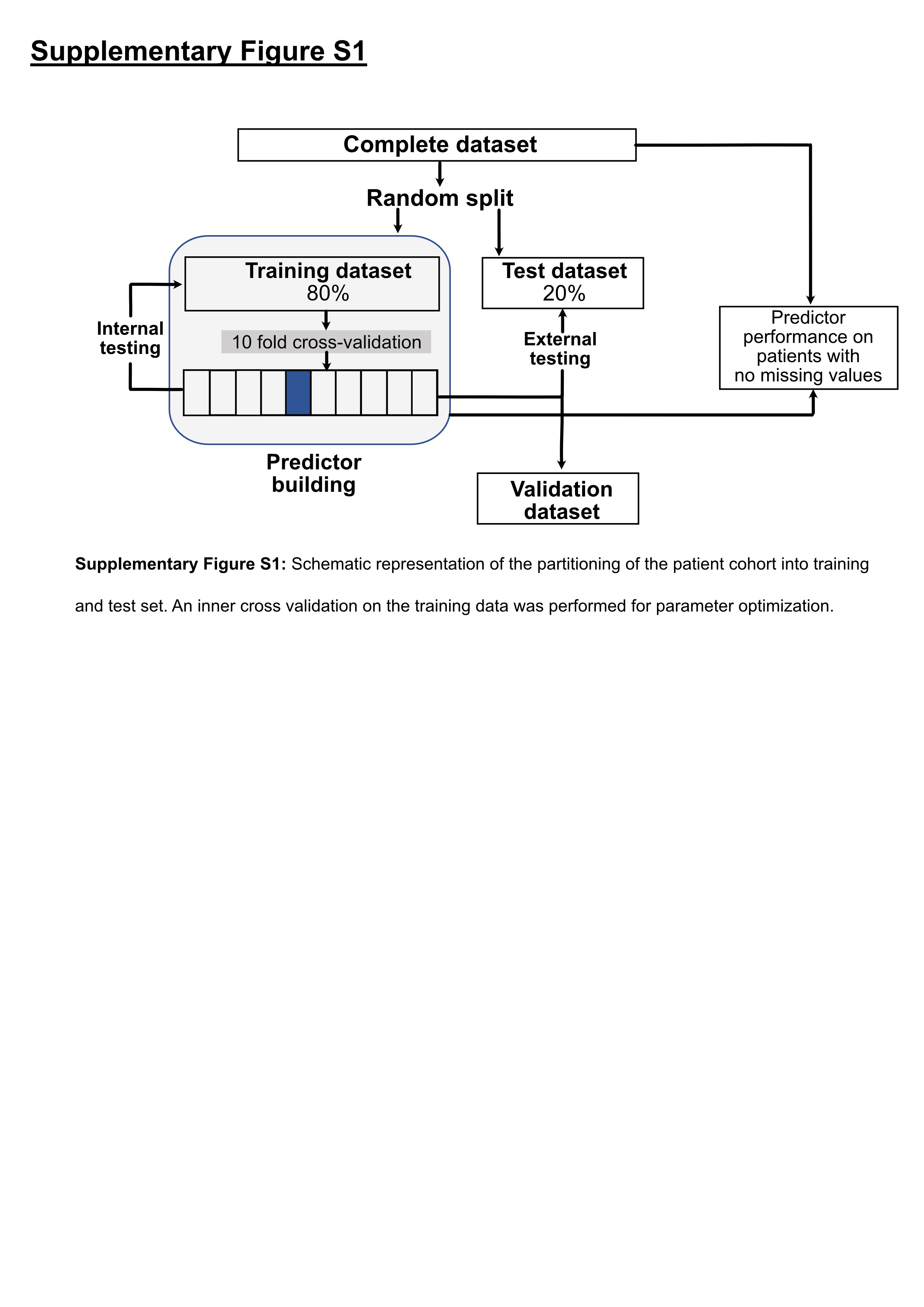

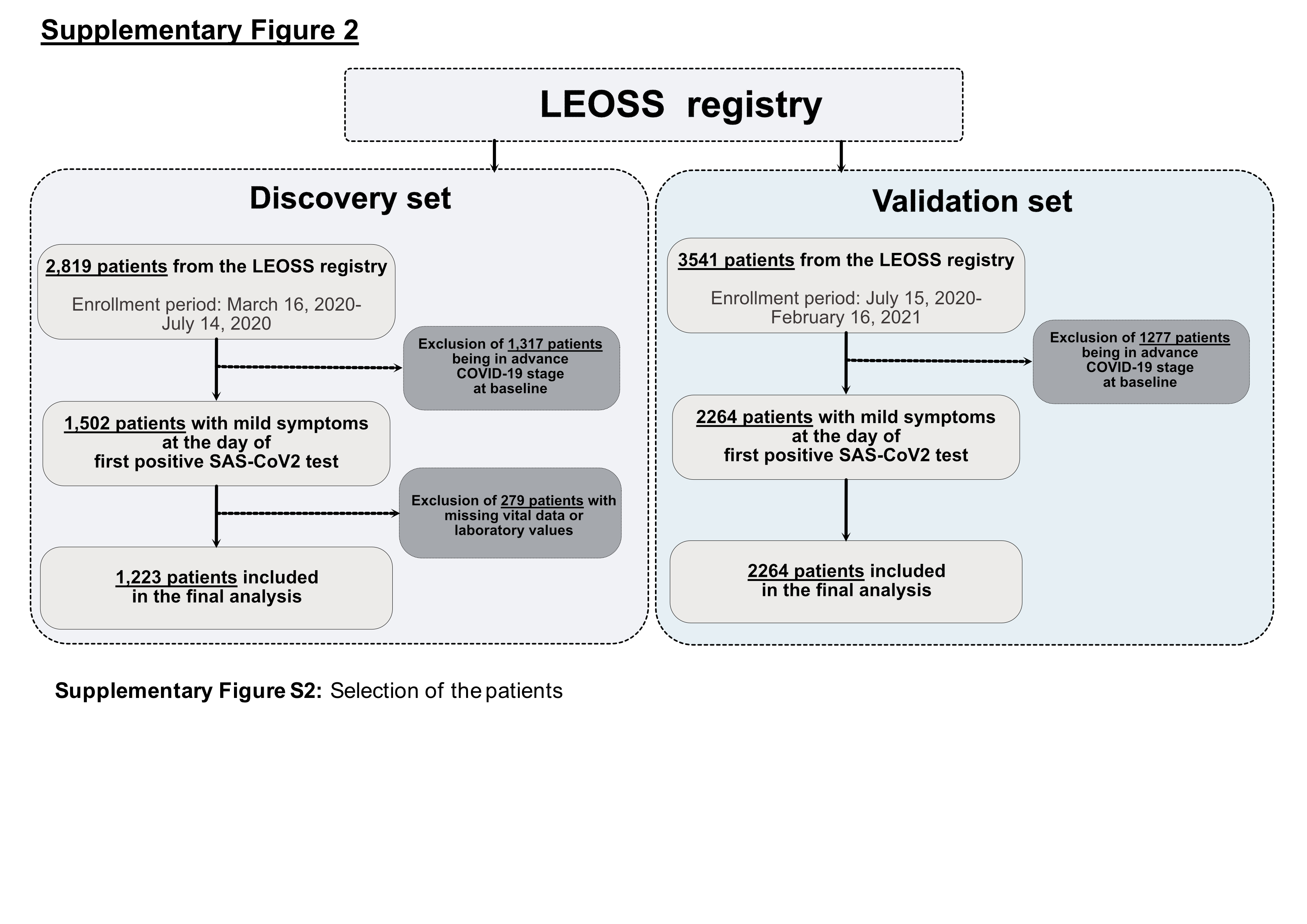

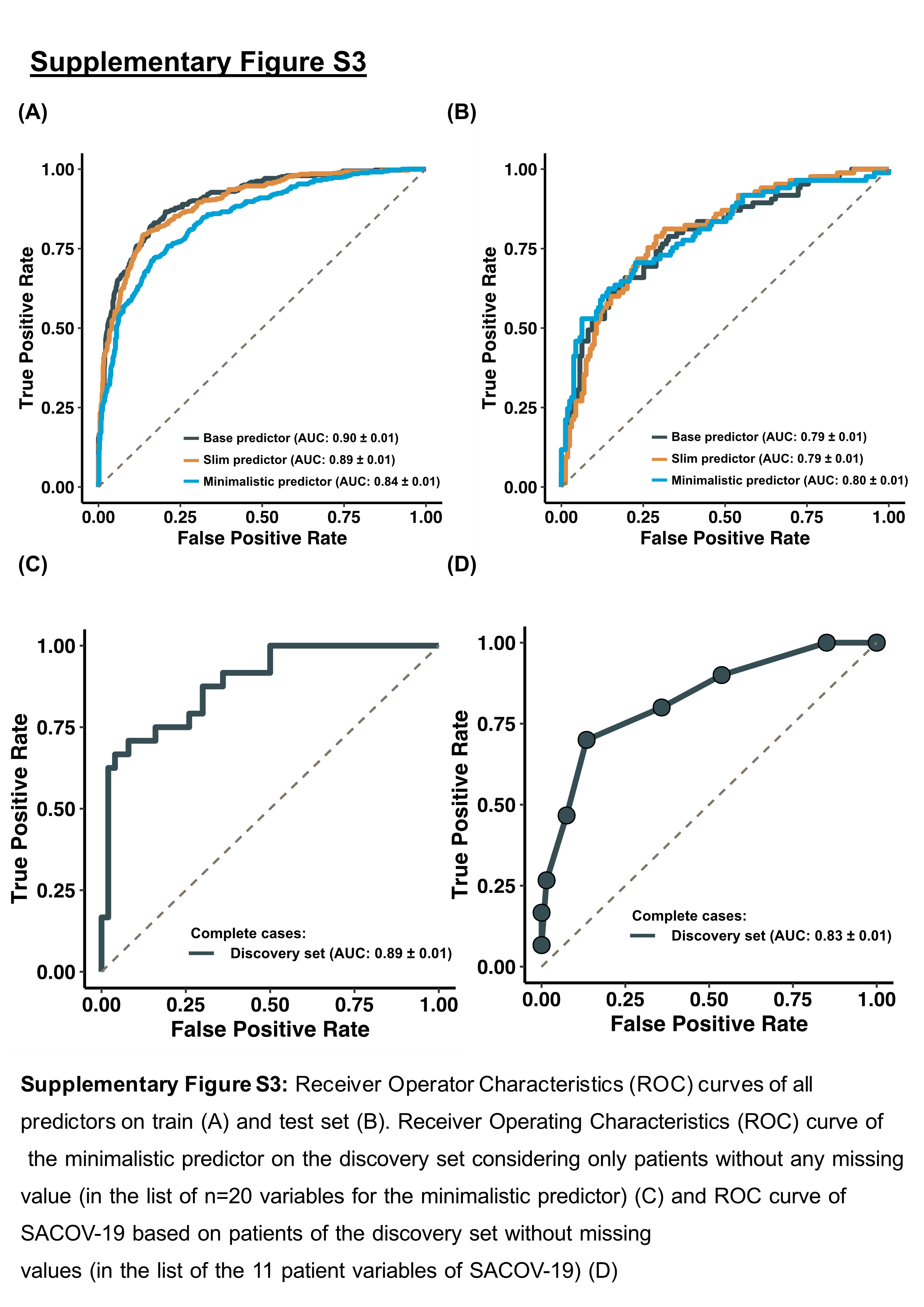

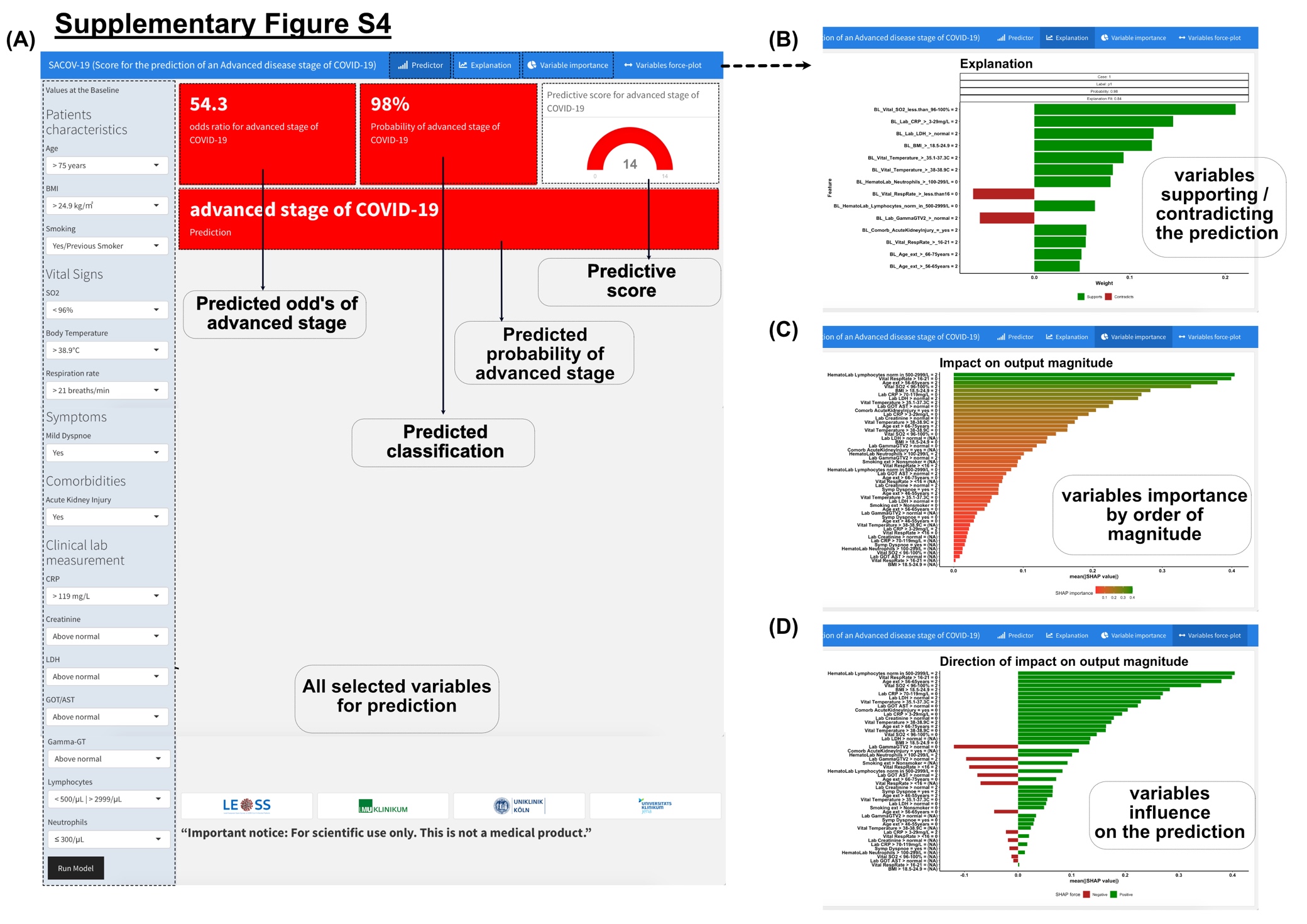


**Supplementary Figure S4:** Screenshot of the graphical user interface of the implementation of the predictor via the web. **(A)** Principle user interface for variable entry and prediction. **(B)** Graphical representation illustrating the supporting or contradicting impact of each variable on the decision. **(C-D)** Graphical representation of the magnitude (C) and direction (D) of the impact of each variable on the decision.

**Supplementary movies**

**Supplementary movie M1:**

[**Web-Application_Demo_Prediction of advanced stage of COVID-19**](http://www.klinikum.uni-muenchen.de/Medizinische-Klinik-und-Poliklinik-II/de/sacov19app/index.html)
